# Spiky follicular mycosis fungoides and hidradenitis suppurativa-like lesions in a patient – complete remission with interferon alpha^☆^^☆☆^

**DOI:** 10.1016/j.abd.2019.04.011

**Published:** 2019-12-18

**Authors:** Mónica Garcia-Arpa, Miguel A. Flores-Terry, Monserrat Franco-Muñoz, Isabel María de Lara-Simón

**Affiliations:** a Department of Dermatology, Hospital General Universitario de Ciudad Real, Ciudad Real, Spain; b Department of Pathology, Hospital General Universitario de Ciudad Real, Ciudad Real, Spain

Dear Editor,

Folliculotropic mycosis fungoides (FMF) is a variant of mycosis fungoides (MF) with distinctive clinicopathologic features, in which the neoplastic T lymphocytes display tropism for the follicular epithelium.^1^ The spectrum of the clinical manifestations is heterogeneous.^1^, ^2^ Among them, acneiform lesions are a common presentation, but hidradenitis suppurativa-like lesions (HSLL) are scarcely described in literature. Moreover, spiky follicular mycosis fungoides is an uncommon clinicopathologic presentation, characterized by multiple hyperkeratotic follicular papules.

A healthy 65-year-old man presented a six-month history of generalized cutaneous lesions, alopecia, and severe itching. Dermatological examination revealed numerous spiky follicular papules and alopecia of the scalp (Fig. 1), with follicular keratotic lesions in trichoscopy. Patchy alopecia of the body hair and multiple millimetric hyperkeratotic spicules the on trunk and limbs were present, giving the sensation of rough skin at palpation. Moreover, HSLL were observed in the axillary area. Palpable lymphadenopathy and visceromegalies were not present. The biopsy of scalp showed an infiltrate of atypical lymphocytes in the follicular epithelium, with epidermotropism (Fig. 2). Immunohistochemically, follicular lymphocytes showed positivity for CD3 and CD4, with partial loss of CD7; CD30 was negative. Molecular analysis of TCR revealed a monoclonal population of lymphocytes. Laboratory tests were within normal limits (blood cell count, Sézary cells, biochemistry, electrophoresis, immunoglobulins, β-2 microglobulin) and no systemic involvement was detected in the body scan. A diagnosis of FMF was made. The patient received interferon alpha (IFN-α, 3,000,000 units three times weekly) and topical clobetasol, achieving complete remission one year later without recurrences after three years of follow-up (Fig. 3).

**Figure 1:**
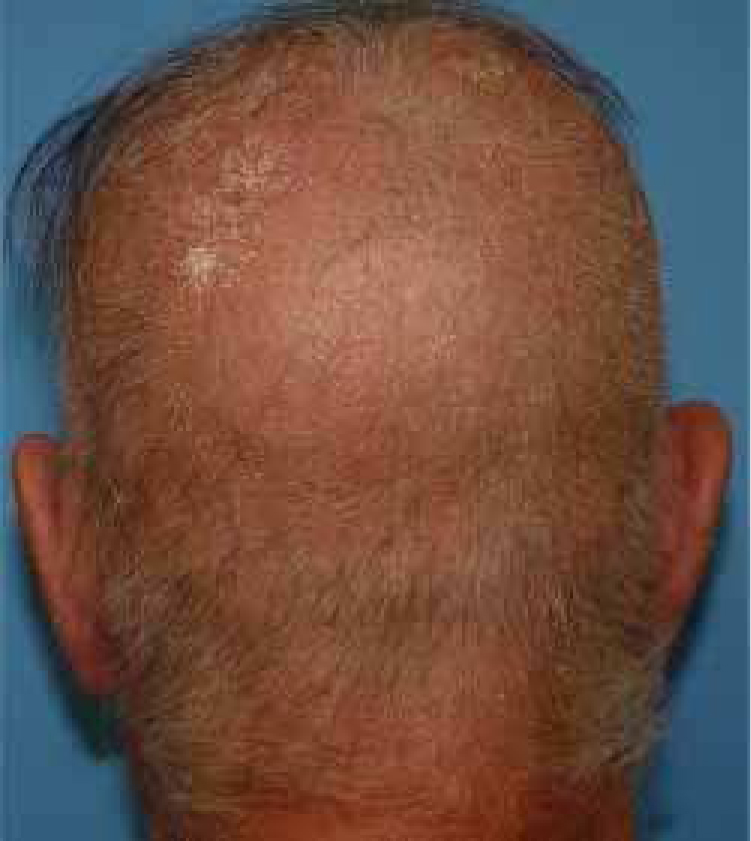
Extensive scalp involvement, with numerous whitish and spiky hyperkeratotic follicular papules, and alopecia.

**Figure 2:**
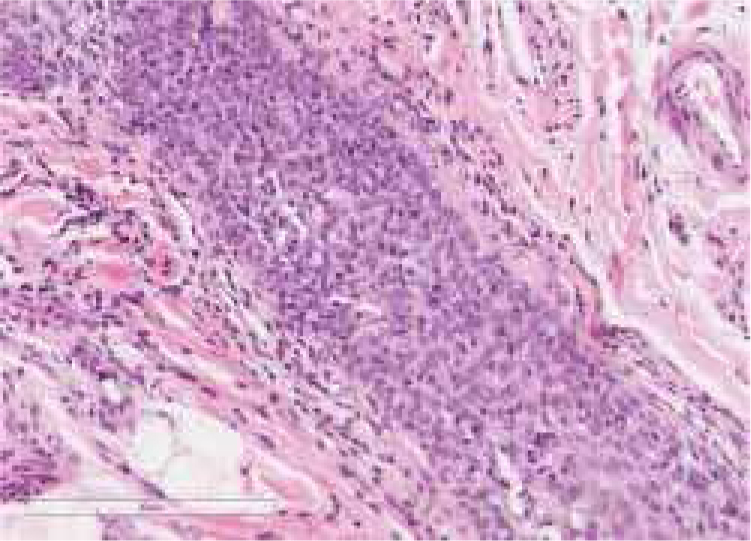
Biopsy of scalp: infiltrate of small-to-medium-sized lymphocytes with mild atypia, around and within follicular epithelium. No follicular mucinosis was present (Hematoxilin & eosin, ×20).

**Figure 3:**
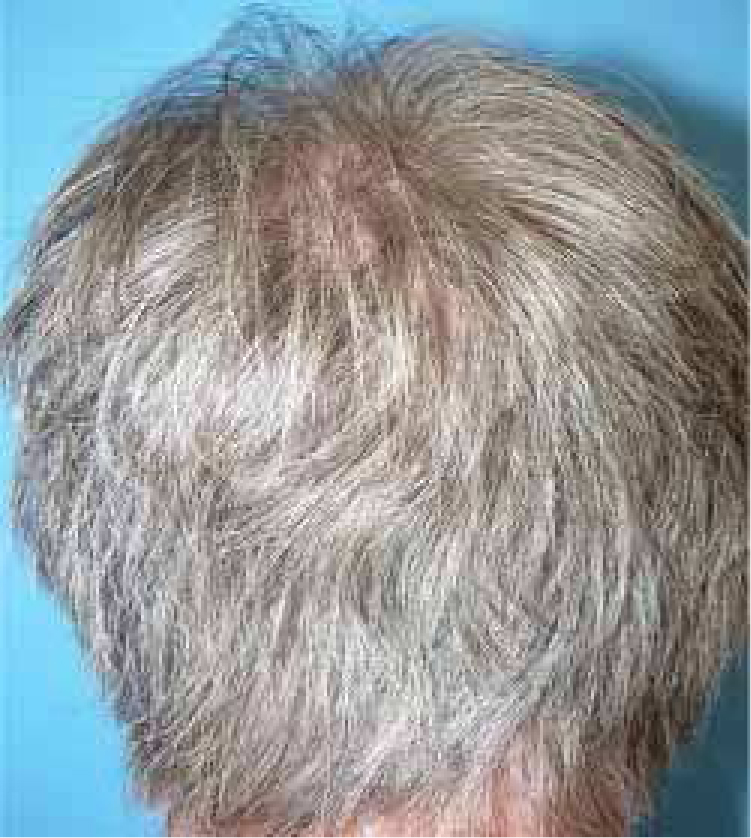
Repopulation of hair after one year of treatment with interferon alpha.

FMF represents less than 10% of patients with MF. This variant is more common in men, with an age of presentation similar to classic forms (around 55–60 years). Typically, it presents as hairless indurated plaques and tumors mainly on the head and neck, with severe pruritus. However, FMF is characterized by a broad clinical spectrum that comprises a variable combination of follicular lesions that may coexist.^1^, ^2^ Among them, spiky FMF has recently been well-described in a series of eight cases.^3^ This peculiar clinical presentation has hardly received attention in the literature, since it is an unusual clinicopathologic presentation of FMF (prevalence of 7.8%).^3^ It represents an early manifestation with a relatively favorable course, especially in the absence of more typical lesions. Clinically, it is characterized by disseminated or localized tiny, hyperkeratotic, spiky and/or cone-shaped follicular papules, giving a rough sensation at palpation. Trichoscopic findings include thick coats of keratinaceous debris around dilated openings and hair shafts, and multiple spicules and keratotic cone-shaped spicules surrounding follicular openings in dermoscopy.^4^ Furthermore, the present case presented axillary HSLL at onset, with nodules and cysts, in the spectrum of acneiform lesions, which are common in FMF. However, HSLL are scarcely mentioned in the literature.

The formation of different follicular lesions in FMF is likely as a result of the extent and degree of infiltration of the hair follicle by the neoplastic infiltrate. The presence of atypical lymphocytes, especially forming collections within the follicular epithelium, is the key feature for the diagnosis. However, the infiltrate may be intermixed with other inflammatory cells and nuclear atypia may be slight, making diagnosis difficult. Moreover, the histopathologic features of hyperkeratotic follicular lesions such as keratosis pilaris like-lesions (KPLL) and spiky FMF may be subtle, with folliculotropic infiltrate of low density, suggestive of early FMF. Furthermore, in spiky FMF, an orthokeratotic or parakeratotic column protruding from the follicular plugging may be observed, and it is remarkable the absence of accompanying inflammatory cells and follicular mucinosis.^3^ Folliculotropic lymphocytes are usually CD4+ (and frequently CD7−) and less commonly CD8+, with occasional T-cell receptor gamma gene rearrangement.

Although the course of FMF is found to be comparable with the tumor stage of classic MF, recent studies indicate a better prognosis for certain patients. Therefore, FMF can be divided into three subgroups considering clinicopathological criteria, with significantly different survival: (1) patients presenting with follicle-based patches and/or follicular papules often associated with alopecia, acneiform lesions, KPLL, or plaques with histologically sparse perifollicular infiltrates, as in the present case, have the best survival and an excellent prognosis (five year and ten year overall survival [OS], 92% and 72%, respectively); (2) patients presenting with infiltrated plaques, histologically characterized by dense perifollicular infiltrates containing many often medium-to-large-sized T cells, tumors, and erythroderma (advanced skin-limited disease) (five year and ten year OS, 55% and 28%); (3) FMF with extracutaneous disease has poor prognosis. Although the optimal treatment for these subgroups needs still to be defined, in the first subgroup, they may benefit from skin-directed therapies.^2^, ^5^

In conclusion, this report described a patient with two unusual manifestations of FMF, with excellent evolution.

## Financial support

None declared.

## Authors’ contribution

Mónica García Arpa: Concept; definition of intellecutal content; literature search; manuscript preparation; manuscript edition; manuscript review.

Miguel A. Flores-Terry: Approval of final version of the manuscript; critical review of the manuscript.

Monserrat Franco-Muñoz: Approval of final version of the manuscript; critical review of the manuscript.

Isabel María de Lara Simón: Approval of final version of the manuscript; critical review of the manuscript.

## Conflicts of interest

None declared.
